# Learning Energy-Based Models in High-Dimensional Spaces with Multiscale Denoising-Score Matching

**DOI:** 10.3390/e25101367

**Published:** 2023-09-22

**Authors:** Zengyi Li, Yubei Chen, Friedrich T. Sommer

**Affiliations:** 1 Redwood Center for Theoretical Neuroscience, Berkeley, CA 94720, USA; yubeic@berkeley.edu (Y.C.); fsommer@berkeley.edu (F.T.S.); 2 Department of Physics, University of California Berkeley, Berkeley, CA 94720, USA; 3 Berkeley AI Research, University of California Berkeley, Berkeley, CA 94720, USA; 4 Helen Wills Neuroscience Institute, University of California Berkeley, Berkeley, CA 94720, USA; 5 Neuromorphic Computing Group, Intel Labs, 2200 Mission College Blvd., Santa Clara, CA 95054, USA

**Keywords:** energy-based model, score matching, generative model

## Abstract

Energy-based models (EBMs) assign an unnormalized log probability to data samples. This functionality has a variety of applications, such as sample synthesis, data denoising, sample restoration, outlier detection, Bayesian reasoning and many more. But, the training of EBMs using standard maximum likelihood is extremely slow because it requires sampling from the model distribution. Score matching potentially alleviates this problem. In particular, denoising-score matching has been successfully used to train EBMs. Using noisy data samples with one fixed noise level, these models learn fast and yield good results in data denoising. However, demonstrations of such models in the high-quality sample synthesis of high-dimensional data were lacking. Recently, a paper showed that a generative model trained by denoising-score matching accomplishes excellent sample synthesis when trained with data samples corrupted with multiple levels of noise. Here we provide an analysis and empirical evidence showing that training with multiple noise levels is necessary when the data dimension is high. Leveraging this insight, we propose a novel EBM trained with multiscale denoising-score matching. Our model exhibits a data-generation performance comparable to state-of-the-art techniques such as GANs and sets a new baseline for EBMs. The proposed model also provides density information and performs well on an image-inpainting task.

## 1. Introduction and Motivation

Treating data as stochastic samples from a probability distribution and developing models that can learn such distributions is at the core of solving a large variety of application problems, such as error correction/denoising [1]; outlier/novelty detection [2,3]; sample generation [4,5]; invariant pattern recognition; Bayesian reasoning [6], which relies on suitable data priors; and many others.

Energy-based models (EBMs) [7,8] assign energy E(x) to each data point x, which implicitly defines a probability density via the Boltzmann distribution $p_{m}\left(x\right)=e^{-E(x)}/Z$. Sampling from this distribution can be used as a generative process that yields plausible samples of x.

Compared to other generative models like GANs [9], flow-based models [10,11] or autoregressive models [12,13], energy-based models have significant advantages. First, they provide explicit (unnormalized) density information, compositionality [14,15], better mode coverage [16] and flexibility [5]. Further, they do not require special model architecture, unlike autoregressive and flow-based models. Recently, energy-based models have been successfully trained with maximum likelihood [4,5], but training can be very computationally demanding due to the need for a sampling model distribution. Variants with a truncated sampling procedure have been proposed, such as contrastive divergence [17]. Such models learn much faster with the drawback of not exploring the state space thoroughly [18].

### Score Matching, Denoising-Score Matching and Deep-Energy Estimators

*Score matching* (SM) [19] circumvents the requirement of sampling the model distribution. In score matching, the score function is defined as the gradient of the log density or the negative energy function. The expected L2 norm of the difference between the model score function and the data score function is minimized.

One convenient way of using score matching is learning the energy function corresponding to a Gaussian kernel Parzen density estimator [20] of the data: $p_{\sigma_{0}}\left(\tilde{x}\right)=\int q_{\sigma_{0}}\left(\tilde{x}|x\right)p\left(x\right)dx$. Though hard to evaluate, the data score is well defined, $s_{d}\left(\tilde{x}\right)=\nabla_{\tilde{x}}\log\left(p_{\sigma_{0}}\left(\tilde{x}\right)\right)$, and the corresponding objective is

(1) $$L_{SM}\left(\theta \right)=E_{p_{\sigma 0}\left(\tilde{x}\right)}{\left\|\nabla_{\tilde{x}}\log\left(p_{\sigma_{0}}\left(\tilde{x}\right)\right)+\nabla_{\tilde{x}}E\left(\tilde{x};\theta \right)\right\|}^{2}$$

$L_{SM}$ is also known as the Fisher divergence or the Fisher information distance [21,22], $L_{SM}=D_{FD}(p_{\sigma 0}\left|\right|p_{m})$, where $p_{m}\left(x\right)=e^{-E(x)}/Z$ is the normalized distribution from the model energy function. While the KL divergence requires the ratio between two density functions, this metric does not depend on the normalizing constant *Z*, which for an energy-based model needs global integration through sampling and is rarely accurately available.

Vincent [23] studied the connection between a denoising autoencoder and score matching and proved the remarkable result that the following objective, named *denoising-score matching* (DSM), is equivalent to the objective above:

(2) $$L_{DSM}\left(\theta \right)=E_{p_{\sigma_{0}}\left(\tilde{x},x\right)}{\left\|\nabla_{\tilde{x}}\log\left(q_{\sigma 0}\left(\tilde{x}|x\right)\right)+\nabla_{\tilde{x}}E\left(\tilde{x};\theta \right)\right\|}^{2}$$

Note that in (2), the Parzen density score is replaced by the derivative of the log density of the single noise kernel $\nabla_{\tilde{x}}\log\left(q_{\sigma_{0}}\left(\tilde{x}|x\right)\right)$, which is much easier to evaluate. In the particular case of Gaussian noise, $\log\left(q_{\sigma_{0}}\left(\tilde{x}|x\right)\right)=-\frac{{\left(\tilde{x}-x\right)}^{2}}{2\sigma_{0}^{2}}+C$, and therefore

(3) $$L_{DSM}\left(\theta \right)=E_{p_{\sigma 0}\left(\tilde{x},x\right)}{\left\|x-\tilde{x}+{\sigma_{0}}^{2}\nabla_{\tilde{x}}E\left(\tilde{x};\theta \right)\right\|}^{2}$$

The intuition behind objective (3) is simple: it forces the energy gradient to align with the vector pointing from the noisy sample to the clean data sample.

To optimize an objective involving the derivative of a function defined by a neural network, Kingma and LeCun [24] proposed double backpropagation [25]. *Deep-energy estimator networks* [26] first applied this technique to learn an energy function defined by a deep neural network. In this work and similarly in Saremi and Hyvärinen [27], an energy-based model was trained to match a Parzen density estimator of data with a particular noise magnitude. The previous models could perform denoising tasks, but they could not generate high-quality data samples from a random input initialization. Recently, Song and Ermon [28] trained an excellent generative model by fitting a series of score estimators coupled together in a single neural network, each matching the score of a Parzen estimator with a different noise magnitude.

The questions we address here are why learning energy-based models with a single noise level does not permit high-quality sample generation and what can be performed to improve such energy-based models. Our work builds on key ideas from Saremi et al. [26], Saremi and Hyvärinen [27] and Song and Ermon [28].

Section 2 provides a geometric view of the learning problem in denoising-score matching and provides a theoretical explanation of why training with one noise level is insufficient if the data dimension is high.

Section 3 presents a novel method for training an energy-based model, *multiscale denoising-score matching* (MDSM). Section 4 describes the empirical results of the MDSM model and comparisons with other models.

## 2. A Geometric View of Denoising-Score Matching

Song and Ermon [28] used denoising-score matching with a range of noise levels, achieving great empirical results. The authors explained that large noise perturbations are required to enable the learning of the score in low-data density regions. But, it is still unclear why a series of different noise levels are necessary, rather than one single noise level that is large enough. Following Saremi and Hyvärinen [27], we analyze the learning process in denoising-score matching based on the measure concentration properties of high-dimensional random vectors.

We adopt the common assumption that the data distribution to be learned is high-dimensional but only has support around a relatively low-dimensional manifold [29,30,31]. If the assumption holds, it causes a problem for score matching: the density, or the gradient of the density, is then undefined outside the manifold, making it difficult to train a valid density model for the data distribution defined on the entire space. Saremi and Hyvärinen [27] and Song and Ermon [28] discussed this problem and proposed to smooth the data distribution with a Gaussian kernel to alleviate the issue.

To further understand the learning in denoising-score matching when the data lie on a manifold X and the data dimension is high, two elementary properties of random Gaussian vectors in high-dimensional spaces are helpful: First, the length distribution of random vectors becomes concentrated at $\sqrt{d}\sigma$ [32], where $\sigma^{2}$ is the variance of a single dimension. Second, a random vector is always close to orthogonal to a fixed vector [33]. With these premises, one can visualize the configuration of noisy and noiseless data points that enter the learning process: A data point x sampled from X and its noisy version $\tilde{x}$ always lie on a line which is almost perpendicular to the tangent space $T_{x}X$ and intersects X at x. Further, the distance vectors between $\left(x,\tilde{x}\right)$ pairs all have a similar length $\sqrt{d}\sigma$. As a consequence, the set of noisy data points concentrates on a set ${\tilde{X}}_{\sqrt{d}\sigma ,\epsilon }$ that has a distance of $\left(\sqrt{d}\sigma -\epsilon ,\sqrt{d}\sigma +\epsilon \right)$ from the data manifold X, where $\epsilon \ll \sqrt{d}\sigma$.

**Figure 1:**
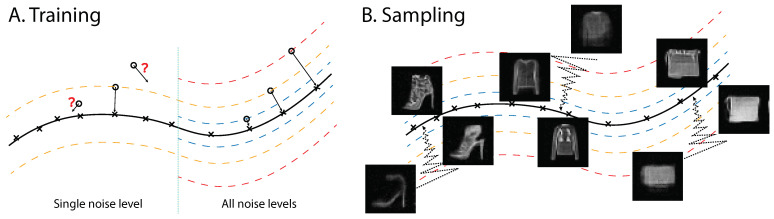
Illustration of multiscale denoising-score matching. (**A**) During training, the derivative of the log likelihood is forced to point toward the data manifold, establishing the energy difference between points within the manifold and outside. Note that energy is negative log likelihood; therefore, energy is higher for points further away from the data manifold. (**B**) During annealed Langevin sampling, the sample travels from the outside data manifold to the data manifold. Single-step denoised samples are shown during sampling of an energy function trained with MDSM on Fashion MNIST (see text for details).

Therefore, performing denoising-score-matching learning with $\left(x,\tilde{x}\right)$ pairs generated with a fixed noise level σ, which is the approach taken previously except in [28], will match the score in the set ${\tilde{X}}_{\sqrt{d}\sigma ,\epsilon }$ and enable the denoising of noisy points in the same set. However, the learning provides little information about the density outside this set, farther or closer to the data manifold, as noisy samples outside ${\tilde{X}}_{\sqrt{d}\sigma ,\epsilon }$ rarely appear in the training process. An illustration is presented in Figure 1A.

Let ${\tilde{X}}_{\sqrt{d}\sigma ,\epsilon }^{C}$ denote the complement of the set ${\tilde{X}}_{\sqrt{d}\sigma ,\epsilon }$. Even if $p_{\sigma_{0}}\left(\tilde{x}\in {\tilde{X}}_{\sqrt{d}\sigma ,\epsilon }^{C}\right)$ is tiny in a high-dimensional space, the score in ${\tilde{X}}_{\sqrt{d}\sigma ,\epsilon }^{C}$ still plays a critical role in sampling from random initialization. This analysis may explain why models based on denoising-score matching, trained with a single noise level, encounter difficulties in generating data samples with random initialization. For empirical support of this explanation, see our experiments with models trained with single noise magnitudes (Appendix B). To remedy this problem, one has to apply a learning procedure proposed in [28], in which samples with different noise levels are used. Depending on the dimension of the data, the different noise levels have to be spaced narrowly enough to avoid empty regions in the data space. In the following, we will use Gaussian noise and employ a Gaussian scale mixture to produce the noisy data samples for the training (for details, see Section 3.1 and Appendix A).

**Figure A1:**
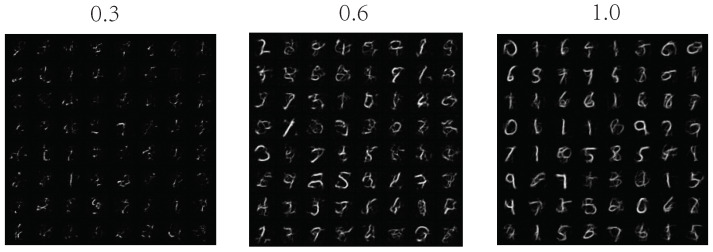
Denoised samples from energy-based model trained with denoising-score matching with single-magnitude Gaussian noise on MNIST. Noise magnitude used in training is shown in above samples. Larger noise scales require the model to learn longer-range correlations in the data distribution, which may explain why samples generated from larger noise scales show more consistent shape.

Another interesting property of denoising-score matching was suggested in the denoising autoencoder literature [1,34]. With an increasing noise level, the learned features tend to have a larger spatial scale. In our experiment, we observe a similar phenomenon when the training model experiences denoising-score matching with a single noise scale. If one compares the samples in Figure A1, Appendix B, it is evident that a noise level of 0.3 produced a model that learned a short-range correlation that spans only a few pixels, a noise level of 0.6 produced a more extended stroke structure without a coherent overall structure and a noise level of 1 produced a more coherent long-range structure without details such as stroke-width variations. This suggests that training with a single noise level for denoising-score matching is insufficient for learning in a model capable of high-quality sample synthesis. For that, a model has to capture data structures at all scales.

## 3. Learning Energy-Based Model with Multiscale Denoising-Score Matching

### 3.1. Multiscale Denoising-Score Matching

Motivated by the analysis in Section 2, we strive to develop an EBM based on denoising-score matching that can be trained with noisy samples in which the noise level is not fixed but drawn from a distribution. The model should approximate the Parzen density estimator of the data $p_{\sigma_{0}}\left(\tilde{x}\right)=\int q_{\sigma_{0}}\left(\tilde{x}|x\right)p\left(x\right)dx$. Specifically, the learning should minimize the difference between the derivative of the energy and the score of $p_{\sigma_{0}}$ under the expectation $E_{p_{M}\left(\tilde{x}\right)}$ rather than $E_{p_{\sigma_{0}}\left(\tilde{x}\right)}$, the expectation taken in standard denoising-score matching. Here, $p_{M}\left(\tilde{x}\right)=\int q_{M}\left(\tilde{x}|x\right)p\left(x\right)dx$ is chosen to cover the signal space more evenly to avoid the measure concentration issue described above. The resulting *multiscale score matching* (MSM) objective is

(4) $$L_{MSM}\left(\theta \right)=E_{p_{M}\left(\tilde{x}\right)}{\left\|\nabla_{\tilde{x}}\log\left(p_{\sigma_{0}}\left(\tilde{x}\right)\right)+\nabla_{\tilde{x}}E\left(\tilde{x};\theta \right)\right\|}^{2}$$

Compared to the objective of denoising-score matching (1), the only change in the new objective (4) is the expectation. Both objectives are consistent if $p_{M}\left(\tilde{x}\right)$ and $p_{\sigma_{0}}\left(\tilde{x}\right)$ have the same support, as shown formally in Proposition A1 of Appendix A. In Proposition A2, we prove that Equation (4) is equivalent to the following denoising-score-matching objective:

(5) $$L_{MDSM^{*}}=E_{p_{M}\left(\tilde{x}\right)q_{\sigma_{0}}\left(x|\tilde{x}\right)}{\left\|\nabla_{\tilde{x}}\log\left(q_{\sigma 0}\left(\tilde{x}|x\right)\right)+\nabla_{\tilde{x}}E\left(\tilde{x};\theta \right)\right\|}^{2}$$

The above results hold for any noise kernel $q_{\sigma_{0}}\left(\tilde{x}|x\right)$, but Equation (5) contains the reversed expectation, which is difficult to evaluate in general. To proceed, we choose $q_{\sigma_{0}}\left(\tilde{x}|x\right)$ to be Gaussian, and also choose $q_{M}\left(\tilde{x}|x\right)$ to be a Gaussian scale mixture: $q_{M}\left(\tilde{x}|x\right)=\int q_{\sigma }\left(\tilde{x}|x\right)p\left(\sigma \right)d\sigma$ and $q_{\sigma }\left(\tilde{x}|x\right)=N\left(x,\sigma^{2}I_{d}\right)$. After algebraic manipulation and one approximation (see the derivation following Proposition A2 in Appendix A), we can transform Equation (5) into a more convenient form, which we call *multiscale denoising-score matching* (MDSM):

(6) $$L_{MDSM}=E_{p\left(\sigma \right)q_{\sigma }\left(\tilde{x}|x\right)p\left(x\right)}{\left\|\nabla_{\tilde{x}}\log\left(q_{\sigma 0}\left(\tilde{x}|x\right)\right)+\nabla_{\tilde{x}}E\left(\tilde{x};\theta \right)\right\|}^{2}$$

The square loss term evaluated at noisy points $\tilde{x}$ at larger distances from the true data points x will have a much larger magnitude. Therefore, in practice, it is necessary to add a monotonically decreasing term l(σ) to balance the loss in different noise scales, e.g., $l\left(\sigma \right)=\frac{1}{\sigma^{2}}$. Ideally, we want our model to learn the correct gradient everywhere, so we need to add noise at all levels. However, learning denoising-score matching at very large or very small noise levels is useless. At huge noise levels, the information of the original sample is completely lost. Conversely, in the limit of small noise, the noisy sample is virtually indistinguishable from real data. In neither case, one can learn an informative gradient about the data structure. Thus, the noise range must only be broad enough to encourage the learning of data features over all scales. Particularly, we do not sample σ but instead choose a series of fixed σ values $\sigma_{1}\cdots \sigma_{K}$. Further, substituting $\log\left(q_{\sigma_{0}}\left(\tilde{x}|x\right)\right)=-\frac{{\left(\tilde{x}-x\right)}^{2}}{2\sigma_{0}^{2}}+C$ into Equation (4), we arrive at the final objective:

(7) $$L\left(\theta \right)=\sum_{\sigma \in \{\sigma_{1}\cdots \sigma_{K}\}}E_{q_{\sigma }\left(\tilde{x}|x\right)p\left(x\right)}l\left(\sigma \right){\left\|x-\tilde{x}+\sigma_{0}^{2}\nabla_{\tilde{x}}E\left(\tilde{x};\theta \right)\right\|}^{2}$$

It may seem that $\sigma_{0}$ is an important hyperparameter for our model. Still, after our approximation, $\sigma_{0}$ becomes just a scaling factor in front of the energy function. It can be set to one as long as the temperature range during sampling is scaled accordingly (see Section 3.2). Therefore, the only hyperparameter is the range of noise levels used during training.

On the surface, Objective (7) looks similar to the one in Song and Ermon [28]. The important difference is that Equation (7) approximates a *single* distribution, namely $p_{\sigma_{0}}\left(\tilde{x}\right)$, the data smoothed with one fixed kernel $q_{\sigma_{0}}\left(\tilde{x}|x\right)$. In contrast, Song and Ermon [28] approximate the score of *multiple* distributions, the family of distributions $\left\{p_{\sigma_{i}}\left(\tilde{x}\right):i=1,...,n\right\}$, resulting from the data smoothed by kernels of different widths $\sigma_{i}$. Because our model learns only a single target distribution, it does not require noise magnitude as the input.

### 3.2. Sampling by Annealed Langevin Dynamics

Langevin dynamics has been used to sample from neural network energy functions [4,5]. However, those studies described difficulties with mode exploration unless many sampling steps were used. We propose incorporating simulated annealing in the Langevin dynamics to improve mode exploration. Simulated annealing [35,36] improves mode exploration by sampling first at a high temperature and then cooling down gradually. This has been successfully applied to challenging computational problems like combinatorial optimization.

To apply simulated annealing to Langevin dynamics, note that in a model of the Brownian motion of a physical particle, the temperature in the Langevin equation enters as a factor $\sqrt{T}$ in front of the noise term; some literature uses $\sqrt{\beta^{-1}}$, where β=1/T [37]. Adopting the $\sqrt{T}$ convention, the Langevin sampling process [38] is given by:

(8) $$x_{t+1}=x_{t}-\frac{\epsilon^{2}}{2}\nabla_{x}E\left(x_{t};\theta \right)+\epsilon \sqrt{T_{t}}N\left(0,I_{d}\right)$$

where $T_{t}$ follows some annealing schedule and ϵ denotes the step length, which is fixed. During sampling, samples behave like physical particles under Brownian motion in a potential field. Because the particles have average energies close to their current thermic energy, they explore the state space at different distances from the data manifold depending on the temperature. Eventually, they settle somewhere on the data manifold. The behavior of the particle’s energy value during a typical annealing process is depicted in Appendix F in Figure A7B.

If the obtained sample is still slightly noisy, we can apply a single-step gradient denoising jump [27] to improve the sample quality:

(9) $$x_{clean}=x_{noisy}-\sigma_{0}^{2}\nabla_{x}E\left(x_{noisy};\theta \right)$$

This denoising procedure can be applied to noisy samples with any level of Gaussian noise because, in our model, the gradient automatically has the correct magnitude to denoise the sample. This process is justified by the Empirical Bayes interpretation of this denoising process, as studied in [27].

Song and Ermon [28] also call their sample-generation process annealed Langevin dynamics. It should be noted that their sampling process does not coincide with Equation (8). Their sampling procedure is best understood as sequentially sampling a series of distributions corresponding to data distribution corrupted by different noise levels.

## 4. Image Modeling Results

**Training and Sampling Details.** The proposed energy-based model is trained on standard image datasets, specifically MNIST, Fashion MNIST, CelebA [39] and CIFAR-10 [40]. During training, we set $\sigma_{0}=0.1$ and train over a noise range of σ∈[0.05,1.2], with the different noise uniformly spaced on the batch dimension. For MNIST and Fashion MNIST, we used geometrically distributed noise in the range [0.1,3]. The weighting factor l(σ) is always set to $1/\sigma^{2}$ to make the square term roughly independent of σ. We use a batch size of 128 and the Adam optimizer with a $5\times 10^{-5}$ learning rate. For MNIST and Fashion MNIST, we use a 12-layer ResNet with 64 filters. For the CelebA and CIFAR-10 datasets, we used a 18-layer ResNet with 128 filters [41,42]. No normalization layer was used in any of the networks. We designed the output layer of all networks to take a generalized quadratic form [43]. Because the energy function is anticipated to be approximately quadratic with respect to the noise level, this modification boosted the performance significantly. For more detail on the training and model architecture, see Appendix D. One notable result is that since our training method does not involve sampling, **we achieved a speed up of roughly an order of magnitude compared to the maximum-likelihood training using Langevin dynamics** (for example, on a single GPU, training MNIST with a 12-layer ResNet takes 0.3 s per batch with our method, while maximum-likelihood training with a modest 30 Langevin steps per weight update takes 3 s per batch. Both methods need a similar number of weight updates to train). Our approach thus enables the training of energy-based models even when limited computational resources prohibit maximum-likelihood methods.

We found that the choice of the maximum noise level has little effect on learning as long as it is large enough to encourage the learning of the longest range features in the data. However, as expected, learning with too small or too large noise levels is not beneficial and can even destabilize the training process. Further, our method appeared to be relatively insensitive to how the noise levels are distributed over a chosen range. Geometrically spaced noise as in [28] and linearly spaced noise both work, although in our case, learning with linearly spaced noise was somewhat more robust.

To sample the learned energy function, we used annealed Langevin dynamics with an annealing schedule where the temperature varies continuously. See Figure A7B for the particular shape of our annealing schedule. In contrast, annealing schedules with a theoretical guaranteed convergence property takes extremely long [44]. The range of temperatures to use in the sampling process depends on the choice of $\sigma_{0}$ as the equilibrium distribution contains rough images with a Gaussian noise of magnitude $\sqrt{T}\sigma_{0}$ added on top. To ease traveling between modes far apart and ensure even sampling, the initial temperature needs to be high enough to inject noise of sufficient magnitude. The choice of T=100, corresponding to added noise of magnitude $\sqrt{100}\times 0.1=1$, is an adequate starting point. For step length ϵ, we generally used 0.02, and [0.015,0.05] appeared to be a reasonable range for this parameter. After annealing, we performed single-step denoising to enhance the sample quality slightly.

**Figure 2:**
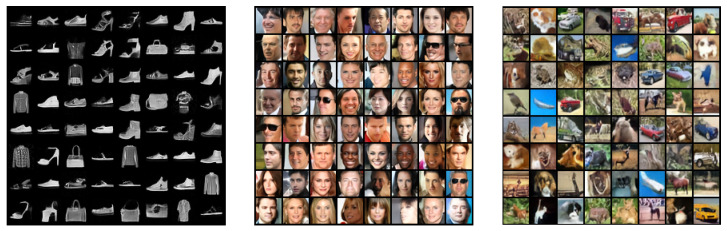
Unconditional samples from our model trained on Fashion MNIST, CelebA and CIFAR-10. See Figure A5 and Figure A6 in Appendix E for more samples and comparison with training data.

**Unconditional Image Generation.** We demonstrate the generative ability of our model by displaying samples obtained by annealed Langevin sampling and single-step denoising jump. We evaluated 50k sampled images after training on CIFAR-10 with two performance scores, Inception [45] and FID [46]. We achieved an Inception Score of 8.31 and an FID of 31.7, comparable to modern GAN approaches. In Figure 2, we display some samples for visual inspection. The scores of the CelebA dataset are not reported here as they are not commonly reported and may depend on the specific preprocessing used. More samples and training images are provided in the Appendix E for visual inspection. We believe the visual assessment is still essential because of the possible issues with the Inception Score [47]. Indeed, we also found that the visually impressive samples were not necessarily the ones achieving the highest Inception Score.

**Figure A2:**
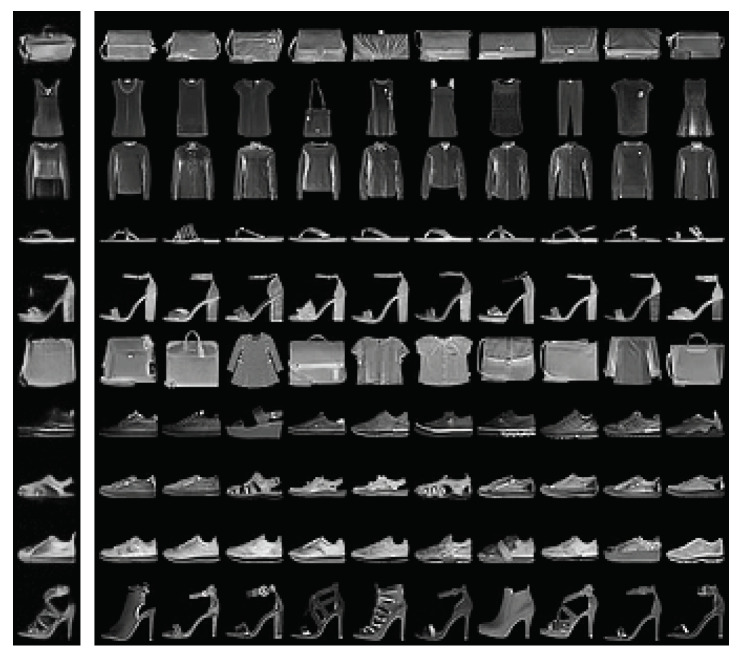
Samples from energy-based model trained on Fashion MNIST (left column) next to 10 (L2) nearest neighbors in the training set.

Although overfitting is not a common concern for generative models, we still tested our model for overfitting. We found no indication of overfitting by comparing the model samples with their nearest neighbors in the dataset. See Figure A2 in Appendix C.

**Mode Coverage.** We repeated, with our model, the three-channel MNIST mode coverage experiment similar to the one in [16]. An energy-based model was trained on three-channel data where each channel is a random MNIST digit. Then, 8000 samples were taken from the model, and each channel was classified by using a small MNIST classifier network. We obtained the results of the 966 modes, comparable to GAN approaches. The training was successful, and our model assigned low energy to all the learned modes. But, some modes were not accessed during sampling, likely due to the Langevin dynamics failing to explore these modes. A better sampling technique such as HMC [48] or a Maximum Entropy Generator [16] could improve this result.

**Figure 3:**
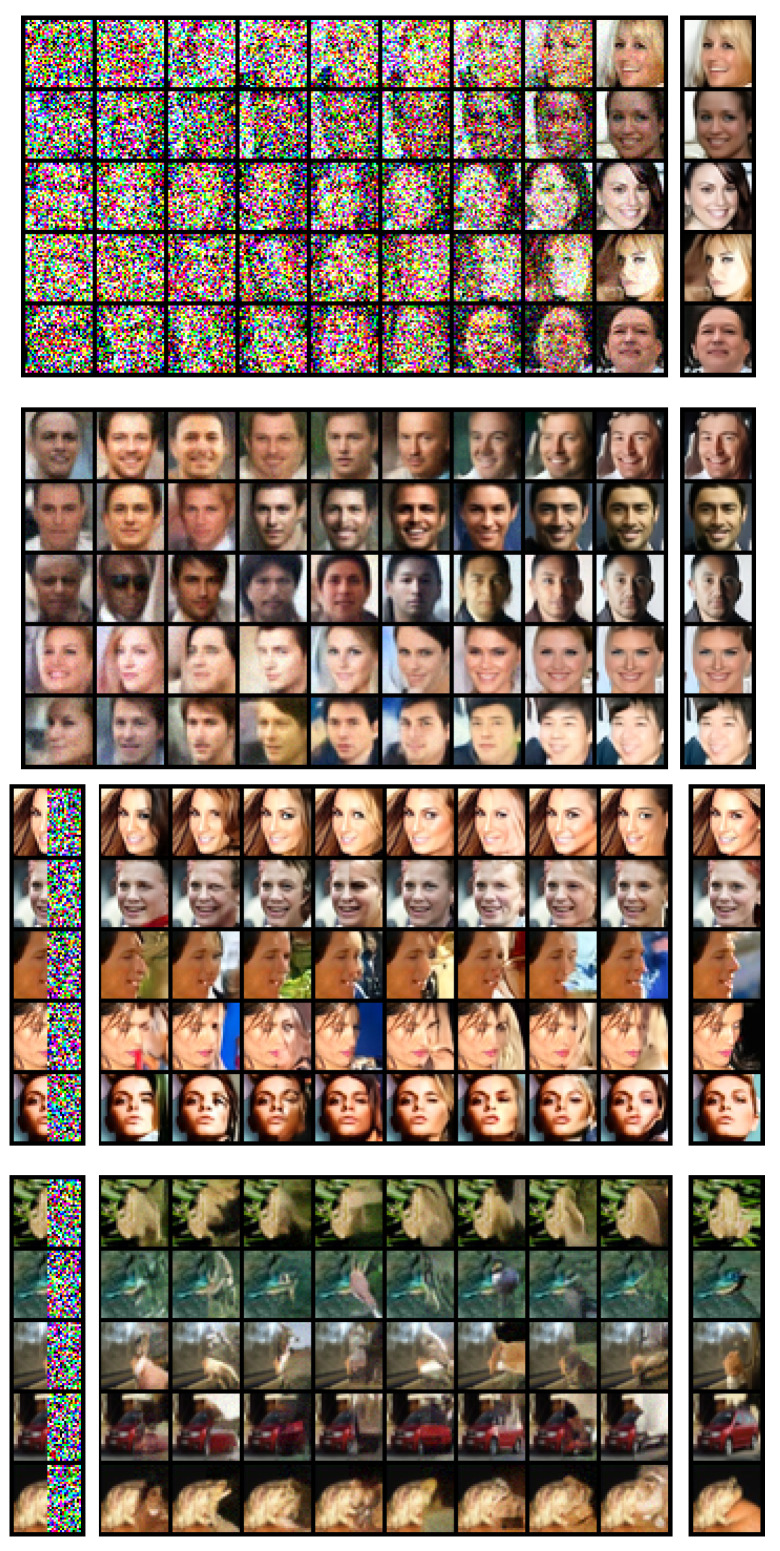
Demonstration of the sampling process (**top two**) and image inpainting (**bottom two**). The sampling process is shown with Gaussian noise (**first**) and denoised by a single-step gradient jump (**second**). The column next to the sampling process shows samples after the last denoising jump at the end of sampling. Inpainting results are shown next to the initial image (**left column**) and the ground-truth image (**right column**).

**Image Inpainting.** Image inpainting can be achieved with our model by clamping a part of the image to ground truth and performing the same annealed Langevin and jump-sampling procedure on the missing part of the image. Noise appropriate for the sampling temperature must be added to the clamped inputs. The quality of the inpainting results of our model trained on CelebA and CIFAR-10 can be assessed in Figure 3. For the CIFAR-10 inpainting results, we used the test set.

**Log-Likelihood Estimation.** For energy-based models, the log density can be obtained after estimating the partition function with Annealed Importance Sampling (AIS) [49] or Reverse AIS [50]. In our experiment on the CIFAR-10 model, similar to reports in [5], there is still a substantial gap between AIS and Reverse AIS estimation, even after significant computational effort. In Table 1, we report the results from Reverse AIS, as it tends to overestimate the partition function, thus underestimating the density. Our reported density value on the CIFAR dataset underperforms compared to other models, likely due to two reasons: The model is approximating a Gaussian kernel density estimator of the data distribution, which is not a very good model on its own. Also, the lower bound obtained by Reverse AIS may not be tight due to the difficulty in sampling.

We also report a density of 6.79 bits/dim on the MNIST dataset, again not comparable to other density models. The density reported here follows the convention of measuring the density of pixel values between [0,255]. More details on this experiment are provided in the Appendix D.

**Outlier Detection.** Choi et al. [3] and Nalisnick et al. [54] reported the intriguing behavior of high-dimensional density models on out-of-distribution samples. Specifically, they showed that many models assign a higher likelihood to out-of-distribution samples than real data samples. We investigated whether our model behaves similarly.

**Figure A7:**
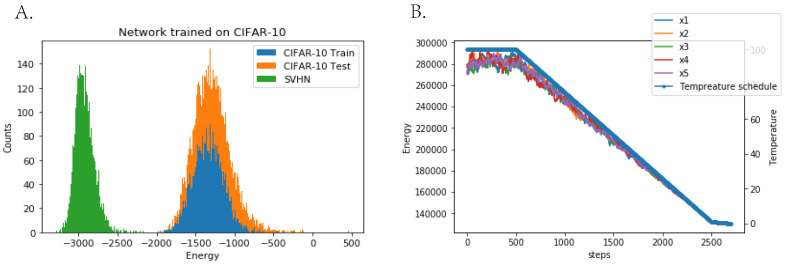
(**A**) Energy values for CIFAR-10 train, CIFAR-10 test and SVHN datasets for a network trained on CIFAR-10 images. Note that the network does not overfit the training set, but just like most deep likelihood models, it assigns lower energy to SVHN images than its own training data. (**B**) Annealing schedule and a typical energy trace for a sample during annealed Langevin sampling. The energy of the sample is proportional to the temperature, indicating sampling is close to a quasistatic process.

Our energy function is only trained outside the data manifold where samples are noisy, so the energy value at clean data points may not always be well-behaved. Therefore, we added noise with magnitude $\sigma_{0}$ before measuring the energy value. Our network behaves similarly to previous likelihood models; it assigns lower energy, and thus a higher density, to some OOD samples. We show one example of this phenomenon in Appendix A in Figure A7.

We also attempted to use the denoising performance, or the objective function, to perform outlier detection. Intriguingly, the results are similar to using the energy value. The denoising performance correlates more with the original image’s variance than the image’s content.

## 5. Discussion

The central goal of our work is to investigate how to build EMBs in high-dimensional spaces with an objective function similar to denoising-score matching or the “Fisher divergence”. We first provided analyses and empirical results for understanding the limitations of learning the structure of high-dimensional data with denoising-score matching. We found that the objective function $L_{SM}$ confines learning to a small set due to the measure concentration phenomenon in high-dimensional random vectors. Therefore, sampling the learned distribution starting from outside the set, where the gradient is learned more accurately, does not produce good results. In our opinion, the expectation with respect to the target distribution $E_{p_{\sigma 0}\left(\tilde{x}\right)}$ is not of critical importance, and it only enforces the score matching in the confined high-probability region. Since sampling from a random initial location requires the model score to match the target distribution score everywhere, we propose that $E_{p_{\sigma 0}\left(\tilde{x}\right)}$ should be replaced by $E_{p_{M}\left(\tilde{x}\right)}$, where $p_{M}\left(\tilde{x}\right)$ covers a much larger range in the signal space than $p_{\sigma 0}\left(\tilde{x}\right)$. This leads to the multiscale denoising-score matching, which can be viewed as “multiscale Fisher Divergence”. The resulting *multiscale denoising-score matching* (MDSM) EBM model can denoise, produce high-quality samples from random noise, perform image inpainting, etc. While also providing density information, our model learns an order of magnitude faster than the models based on maximum likelihood and sampling.

Previous efforts to learn energy-based models with score matching [24,55] cannot produce high-quality samples and sometimes are computationally intensive. Saremi et al. [26] and Saremi and Hyvärinen [27] trained energy-based models with the denoising-score-matching objective. Their method is computationally efficient, but the resulting models cannot perform sample synthesis from random noise initialization. The NCSN recently proposed by Song and Ermon [28] is not an EBM, but it is capable of high-quality sample synthesis. This model learns a sequence of score functions. Each approximates the data distribution smoothed by a different-sized Gaussian. Sample generation in NCSN is achieved by sequential sampling from this set of distributions. Our MDSM method instead learns an energy-based model corresponding to $p_{\sigma_{0}}\left(\tilde{x}\right)$ for a fixed $\sigma_{0}$. This method improves score matching in high-dimensional space by matching the gradient of an energy function to the score of $p_{\sigma_{0}}\left(\tilde{x}\right)$ in the whole space and avoids measure-concentration issues.

All told, we offer a novel EBM model that achieves high-quality sample synthesis, which provides a new state-of-the-art approach among EBM approaches. Compared to the NCSN model, our model is more parsimonious and can support single-step denoising without prior knowledge of the noise magnitude. Our model performs slightly worse than the NCSN model in terms of sample quality, which could have several reasons. First, the derivation of Equation (6) requires an approximation to keep the training procedure tractable, which could be inaccurate. Second, the NCSN’s output is a vector that, at least during optimization, does not always have to be the derivative of a scalar function. In contrast, in our model, the network output is scalar. Thus, the NCSN model may perform better because it explores a more extensive set of functions during optimization. 

## Figures and Tables

**Table 1:** Unconditional Inception Score, FID scores and likelihoods for CIFAR-10. Arrow indicates the better direction of the score.

Model	IS ↑	FID ↓	Likelihood	NLL (bits/dim) ↓
iResNet [51]	-	65.01	Yes	3.45
PixelCNN [12]	4.60	65.93	Yes	**3.14**
PixelIQN [13]	5.29	49.46	Yes	-
Residual Flow [52]	-	46.37	Yes	3.28
GLOW [11]	-	46.90	Yes	3.35
EBM (ensemble) [5]	6.78	38.2	Yes	- ^1^
MDSM (Ours)	8.31	31.7	Yes	7.04 ^2^
SNGAN [53]	8.22	**21.7**	No	-
NCSN [28]	**8.91**	25.32	No	-

^1^ Author-reported difficulties evaluating likelihood. ^2^ Upper bound obtained by Reverse AIS.

**Figure A3:**
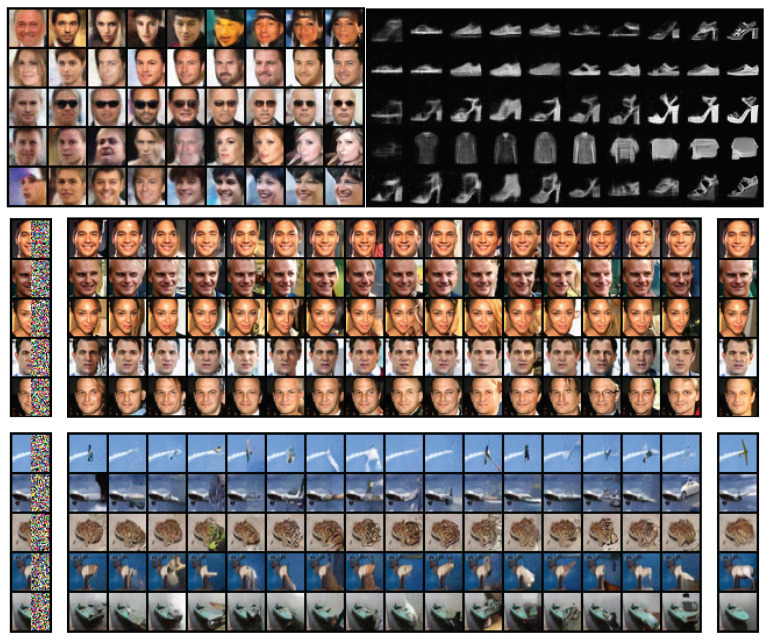
Denoised sampling process and inpainting results. Sampling process is from left to right.

**Figure A4:**
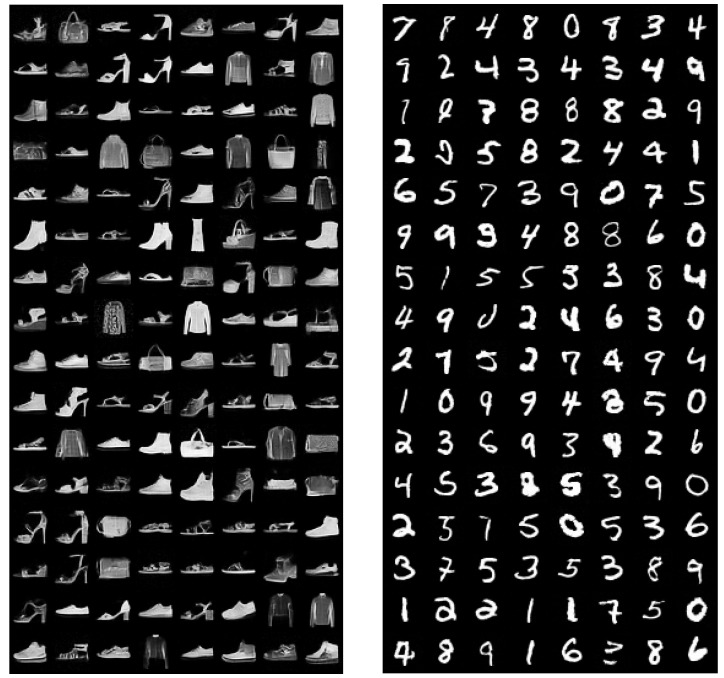
Extended Fashion MNIST and MNIST samples.

**Figure A5:**
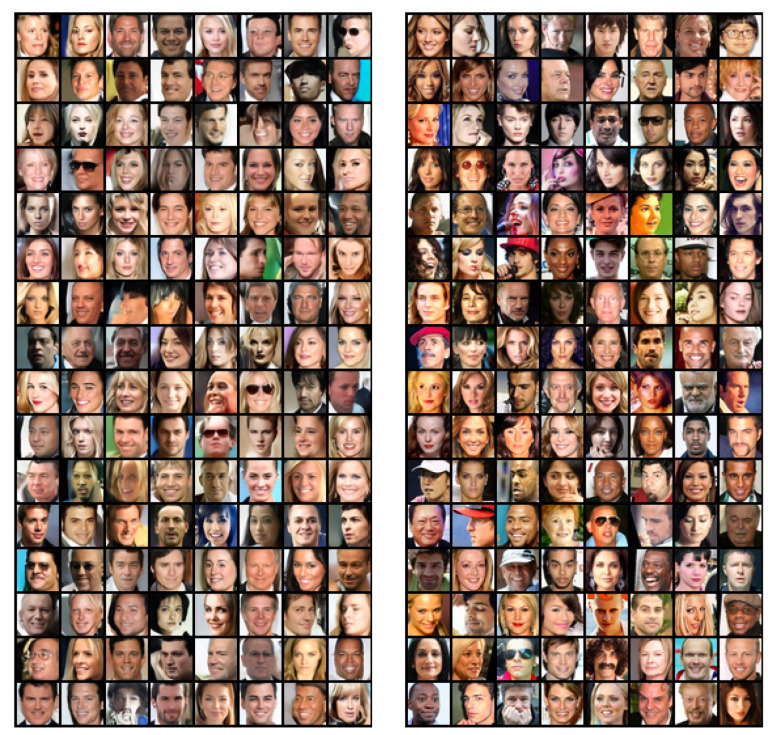
Samples (**left panel**) from network trained on CelebA, and training examples from the dataset (**right panel**).

**Figure A6:**
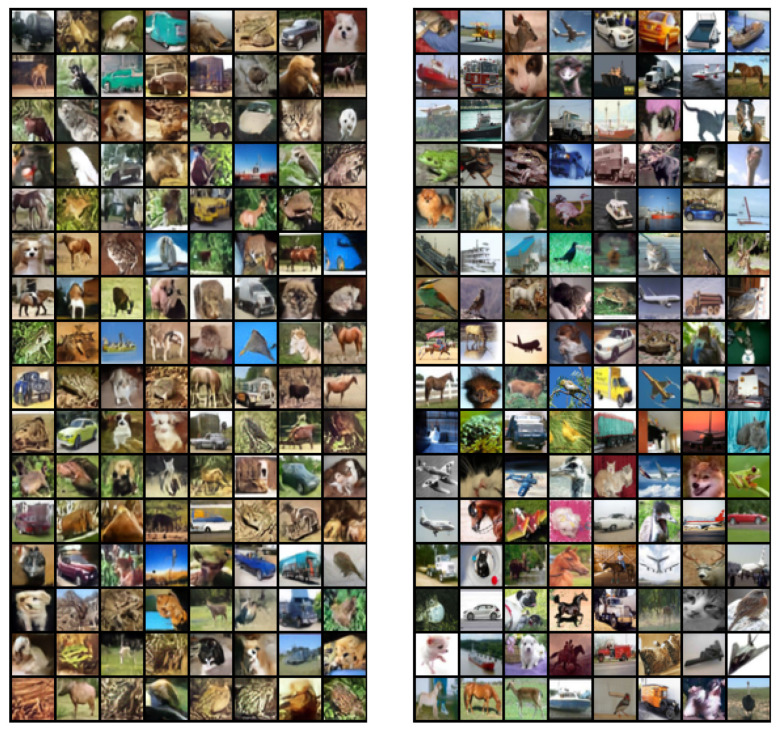
Samples (**left panel**) from energy-based model trained on CIFAR-10 next to training examples (**right panel**).

## Data Availability

This work only used a publicly available dataset. Our code is available at https://github.com/zengyi-li/MDSM.

## Abbreviations

The following abbreviations are used in this manuscript:

EBM	Energy-based model
GAN	Generative Adversarial Networks
SM	Score matching
MDSM	Three-letter acronym
MNIST	Modified National Institute of Standards and Technology database
CelebA	CelebFaces Attributes Dataset
CIFAR-10	Tiny image classification dataset https://www.cs.toronto.edu/~kriz/cifar.html, accessed on March 2019
ResNet	Residual Networks
HMC	Hamiltonian Monte Carlo
AIS	Annealed Importance Sampling
OOD	Out of distribution
NCSN	Noise Conditional Score Networks

## Appendix A. MDSM Objective

In this section, we provide a formal discussion of the MDSM objective and suggest it as an improved score-matching formulation in high-dimensional space.

Vincent [23] illustrated the connection between the model score $-\nabla_{\tilde{x}}E\left(\tilde{x};\theta \right)$ and the score of the Parzen window density estimator $\nabla_{\tilde{x}}\log\left(p_{\sigma_{0}}\left(\tilde{x}\right)\right)$. Specifically, the objective is Equation (1), which we restate here:

(A1) $$L_{SM}\left(\theta \right)=E_{p_{\sigma 0}\left(\tilde{x}\right)}{\left\|\nabla_{\tilde{x}}\log\left(p_{\sigma_{0}}\left(\tilde{x}\right)\right)+\nabla_{\tilde{x}}E\left(\tilde{x};\theta \right)\right\|}^{2}$$

Our key observation is: in high-dimensional space, due to the concentration of measure, the expectation with respect to $p_{\sigma 0}\left(\tilde{x}\right)$ overweighs a thin shell at a rough distance of $\sqrt{d}\sigma$ to the empirical distribution p(x). Though this is not a problem in theory, in practice, this results in the score being only well matched on this shell. Based on this observation, we suggest replacing the expectation with respect to $p_{\sigma 0}\left(\tilde{x}\right)$ with a distribution $p_{\sigma^{\prime }}\left(\tilde{x}\right)$ that has the same support as $p_{\sigma 0}\left(\tilde{x}\right)$ but can avoid the measure concentration problem. We call this *multiscale score matching*, and the objective is the following:

(A2) $$L_{MSM}\left(\theta \right)=E_{p_{M}\left(\tilde{x}\right)}{\left\|\nabla_{\tilde{x}}\log\left(p_{\sigma_{0}}\left(\tilde{x}\right)\right)+\nabla_{\tilde{x}}E\left(\tilde{x};\theta \right)\right\|}^{2}$$

**Proposition** **A1.**
*$L_{MSM}\left(\theta \right)=0\Leftrightarrow L_{SM}\left(\theta \right)=0\Leftrightarrow \theta =\theta^{*}$.*

**Proof.** Given that $p_{M}\left(\tilde{x}\right)$ and $p_{\sigma 0}\left(\tilde{x}\right)$ have the same support, it is clear that $L_{MSM}=0$ would be equivalent to $L_{SM}=0$. Due to the proof of Theorem 2 in Hyvärinen [19], we have $L_{SM}\left(\theta \right)\Leftrightarrow \theta =\theta^{*}$. Thus, $L_{MSM}\left(\theta \right)=0\Leftrightarrow \theta =\theta^{*}$. □

**Proposition** **A2.**
*$L_{MSM}\left(\theta \right)⌣L_{MDSM^{*}}=E_{p_{M}\left(\tilde{x}\right)q_{\sigma 0}\left(x|\tilde{x}\right)}{\left\|\nabla_{\tilde{x}}\log\left(q_{\sigma 0}\left(\tilde{x}|x\right)\right)+\nabla_{\tilde{x}}E\left(\tilde{x};\theta \right)\right\|}^{2}$.*

**Proof.** We follow the same procedure as Vincent [23] to prove this result.

$$\begin{array}{cc} J_{MSM}\left(\theta \right) & =E_{p_{M}\left(\tilde{x}\right)}{\left\|\nabla_{\tilde{x}}\log\left(p_{\sigma_{0}}\left(\tilde{x}\right)\right)+\nabla_{\tilde{x}}E\left(\tilde{x};\theta \right)\right\|}^{2}\\ \; & =E_{p_{M}\left(\tilde{x}\right)}{\left\|\nabla_{\tilde{x}}E\left(\tilde{x};\theta \right)\right\|}^{2}+2S\left(\theta \right)+C \end{array}$$

$$\begin{array}{cc} S(\theta ) & =E_{p_{M}\left(\tilde{x}\right)}\langle \nabla_{\tilde{x}}\log\left(p_{\sigma_{0}}\left(\tilde{x}\right)\right),\nabla_{\tilde{x}}E\left(\tilde{x};\theta \right)\rangle \\ \; & =\int_{\tilde{x}}p_{M}\left(\tilde{x}\right)\langle \nabla_{\tilde{x}}\log\left(p_{\sigma_{0}}\left(\tilde{x}\right)\right),\nabla_{\tilde{x}}E\left(\tilde{x};\theta \right)\rangle \;d\tilde{x}\\ \; & =\int_{\tilde{x}}p_{M}\left(\tilde{x}\right)\langle \frac{\nabla_{\tilde{x}}p_{\sigma_{0}}\left(\tilde{x}\right)}{p_{\sigma_{0}}\left(\tilde{x}\right)},\nabla_{\tilde{x}}E\left(\tilde{x};\theta \right)\rangle \;d\tilde{x}\\ \; & =\int_{\tilde{x}}\frac{p_{M}\left(\tilde{x}\right)}{p_{\sigma_{0}}\left(\tilde{x}\right)}\langle \nabla_{\tilde{x}}p_{\sigma_{0}}\left(\tilde{x}\right),\nabla_{\tilde{x}}E\left(\tilde{x};\theta \right)\rangle \;d\tilde{x}\\ \; & =\int_{\tilde{x}}\frac{p_{M}\left(\tilde{x}\right)}{p_{\sigma_{0}}\left(\tilde{x}\right)}\langle \nabla_{\tilde{x}}\int_{x}p\left(x\right)q_{\sigma_{0}}\left(\tilde{x}|x\right)dx,\nabla_{\tilde{x}}E\left(\tilde{x};\theta \right)\rangle \;d\tilde{x}\\ \; & =\int_{\tilde{x}}\frac{p_{M}\left(\tilde{x}\right)}{p_{\sigma_{0}}\left(\tilde{x}\right)}\langle \int_{x}p\left(x\right)\nabla_{\tilde{x}}q_{\sigma_{0}}\left(\tilde{x}|x\right)dx,\nabla_{\tilde{x}}E\left(\tilde{x};\theta \right)\rangle \;d\tilde{x}\\ \; & =\int_{\tilde{x}}\frac{p_{M}\left(\tilde{x}\right)}{p_{\sigma_{0}}\left(\tilde{x}\right)}\langle \int_{x}p\left(x\right)q_{\sigma_{0}}\left(\tilde{x}|x\right)\nabla_{\tilde{x}}\log q_{\sigma_{0}}\left(\tilde{x}|x\right)dx,\nabla_{\tilde{x}}E\left(\tilde{x};\theta \right)\rangle \;d\tilde{x}\\ \; & =\int_{\tilde{x}}\int_{x}\frac{p_{M}\left(\tilde{x}\right)}{p_{\sigma_{0}}\left(\tilde{x}\right)}p\left(x\right)q_{\sigma_{0}}\left(\tilde{x}|x\right)\langle \nabla_{\tilde{x}}\log q_{\sigma_{0}}\left(\tilde{x}|x\right),\nabla_{\tilde{x}}E\left(\tilde{x};\theta \right)\rangle \;d\tilde{x}dx\\ \; & =\int_{\tilde{x}}\int_{x}\frac{p_{M}\left(\tilde{x}\right)}{p_{\sigma_{0}}\left(\tilde{x}\right)}p_{\sigma_{0}}\left(\tilde{x},x\right)\langle \nabla_{\tilde{x}}\log q_{\sigma_{0}}\left(\tilde{x}|x\right),\nabla_{\tilde{x}}E\left(\tilde{x};\theta \right)\rangle \;d\tilde{x}dx\\ \; & =\int_{\tilde{x}}\int_{x}p_{M}\left(\tilde{x}\right)q_{\sigma_{0}}\left(x|\tilde{x}\right)\langle \nabla_{\tilde{x}}\log q_{\sigma_{0}}\left(\tilde{x}|x\right),\nabla_{\tilde{x}}E\left(\tilde{x};\theta \right)\rangle \;d\tilde{x}dx \end{array}$$

Thus, we have:

$$\begin{array}{cc} L_{MSM}\left(\theta \right) & =E_{p_{M}\left(\tilde{x}\right)}{\left\|\nabla_{\tilde{x}}E\left(\tilde{x};\theta \right)\right\|}^{2}+2S\left(\theta \right)+C\\ \; & =E_{p_{M}\left(\tilde{x}\right)q_{\sigma_{0}}\left(x|\tilde{x}\right)}{\left\|\nabla_{\tilde{x}}E\left(\tilde{x};\theta \right)\right\|}^{2}+2E_{p_{M}\left(\tilde{x}\right)q_{\sigma_{0}}\left(x|\tilde{x}\right)}\langle \nabla_{\tilde{x}}\log q_{\sigma_{0}}\left(\tilde{x}|x\right),\nabla_{\tilde{x}}E\left(\tilde{x};\theta \right)\rangle +C\\ \; & =E_{p_{M}\left(\tilde{x}\right)q_{\sigma 0}\left(x|\tilde{x}\right)}{\left\|\nabla_{\tilde{x}}\log\left(q_{\sigma 0}\left(\tilde{x}|x\right)\right)+\nabla_{\tilde{x}}E\left(\tilde{x};\theta \right)\right\|}^{2}+C^{\prime } \end{array}$$

So $L_{MSM}\left(\theta \right)⌣L_{MDSM^{*}}$. □

The above analysis applies to any noise distribution, not limited to Gaussian. But, $L_{MDSM^{*}}$ has a reversed expectation form that is difficult to work with. To proceed further, we study the case where $q_{\sigma_{0}}\left(\tilde{x}|x\right)$ is Gaussian and choose $q_{M}\left(\tilde{x}|x\right)$ as a Gaussian scale mixture and $p_{M}\left(\tilde{x}\right)=\int q_{M}\left(\tilde{x}|x\right)p\left(x\right)dx$. By Propositions A1 and A2, we have the following form to optimize:

(A3) $$\begin{array}{cc} L_{MDSM^{*}}\left(\theta \right) & =\int_{\tilde{x}}\int_{x}p_{M}\left(\tilde{x}\right)q_{\sigma_{0}}\left(x|\tilde{x}\right){\left\|\nabla_{\tilde{x}}\log\left(q_{\sigma 0}\left(\tilde{x}|x\right)\right)+\nabla_{\tilde{x}}E\left(\tilde{x};\theta \right)\right\|}^{2}d\tilde{x}dx\\ \; & =\int_{\tilde{x}}\int_{x}\frac{q_{\sigma_{0}}\left(x|\tilde{x}\right)}{q_{M}\left(x|\tilde{x}\right)}p_{M}\left(\tilde{x}\right)q_{M}\left(x|\tilde{x}\right){\left\|\nabla_{\tilde{x}}\log\left(q_{\sigma 0}\left(\tilde{x}|x\right)\right)+\nabla_{\tilde{x}}E\left(\tilde{x};\theta \right)\right\|}^{2}d\tilde{x}dx\\ \; & =\int_{\tilde{x}}\int_{x}\frac{q_{\sigma_{0}}\left(x|\tilde{x}\right)}{q_{M}\left(x|\tilde{x}\right)}p_{M}\left(x,\tilde{x}\right){\left\|\nabla_{\tilde{x}}\log\left(q_{\sigma 0}\left(\tilde{x}|x\right)\right)+\nabla_{\tilde{x}}E\left(\tilde{x};\theta \right)\right\|}^{2}d\tilde{x}dx\\ \; & =\int_{\tilde{x}}\int_{x}\frac{q_{\sigma_{0}}\left(x|\tilde{x}\right)}{q_{M}\left(x|\tilde{x}\right)}q_{M}\left(\tilde{x}|x\right)p\left(x\right){\left\|\nabla_{\tilde{x}}\log\left(q_{\sigma 0}\left(\tilde{x}|x\right)\right)+\nabla_{\tilde{x}}E\left(\tilde{x};\theta \right)\right\|}^{2}d\tilde{x}dx\\ \; & \approx L_{MDSM}\left(\theta \right) \end{array}$$

To minimize Equation (A3), we can use the following importance-sampling procedure [56]: we can sample from the empirical distribution p(x), then sample the Gaussian scale mixture $q_{M}\left(\tilde{x}|x\right)$ and finally weight the sample by $\frac{q_{\sigma_{0}}\left(x|\tilde{x}\right)}{q_{M}\left(x|\tilde{x}\right)}$. We expect the ratio to be close to one for the following reasons: Using Bayes rule, $q_{\sigma_{0}}\left(x|\tilde{x}\right)=\frac{p\left(x\right)q_{\sigma_{0}}\left(\tilde{x}|x\right)}{p_{\sigma_{0}}\left(\tilde{x}\right)}$, we can see that $q_{\sigma_{0}}\left(x|\tilde{x}\right)$ only has support on discrete data points x, and the same thing holds for $q_{M}\left(x|\tilde{x}\right)$. Because $\tilde{x}$ is generated by adding Gaussian noise to real data samples, both estimators should give results highly concentrated on the original sample point x. Therefore, in practice, we ignore the weighting factor and use Equation (6). Improving upon this approximation is left for future work.

## Appendix B. Problem with Single-Noise Denoising-Score Matching

To compare with the previous method, we trained an energy-based model with denoising-score matching by using one noise level on MNIST, initialized the sampling with Gaussian noise of the same level, sampled with Langevin dynamics at T=1 for 1000 steps and performed one denoise jump to recover the model’s best estimate of the clean sample, see Figure A1. We used the same 12-layer ResNet as other MNIST experiments. The models were trained for 100,000 steps before sampling.

## Appendix C. Overfitting Test

We demonstrate that the model does not simply memorize training examples by comparing model samples with their nearest neighbors in the training set. We use Fashion MNIST for this demonstration because overfitting can occur easier than on more complicated datasets, see Figure A2.

## Appendix D. Details on Training and Sampling

We used a custom-designed ResNet architecture for all experiments. For MNIST and Fashion MNIST, we used a 12-layer ResNet with 64 filters on the first layer, while for the CelebA and CIFAR datasets, we used an 18-layer ResNet with 128 filters on the first layer. All networks used the ELU activation function. We did not use any normalization in the ResBlocks, and the filer number is doubled at each downsampling block. Details about our networks’ structure can be found in our code release (https://github.com/zengyi-li/ADSM). All the mentioned models can be trained on two GPUs within two days.

Since the gradient of our energy model scales linearly with the noise, we expected our energy function to scale quadratically with noise magnitude. Therefore, we modified the standard energy-based network output layer to take a flexible quadratic form [43]:

(A4) $$E_{out}=\left(\sum_{i}a_{i}h_{i}+b_{1}\right)\left(\sum_{i}c_{i}h_{i}+b_{2}\right)+\sum_{i}d_{i}h_{i}^{2}+b_{3}$$

where $a_{i}$, $c_{i}$, $d_{i}$ and $b_{1},b_{2},b_{3}$ are learnable parameters, and $h_{i}$ is the (flattened) output of the last residual block. We found this modification to significantly improve performance compared to using a simple linear last layer.

For the CIFAR and CelebA results, we trained for 300k weight updates, saving a checkpoint every 5000 updates. We then took 1000 samples from each network checkpoint and used the network with the lowest FID score. For MNIST and Fashion MNIST, we trained for 100k updates and used the last checkpoint. During training, we padded MNIST and Fashion MNIST to 32 × 32 for convenience and randomly flipped the CelebA images. No other modification was performed. We only constrained the gradient of the energy function; the energy value itself could, in principle, be unbounded. However, they naturally stabilized, so we did not explicitly regularize them. The annealing sampling schedule is optimized to improve the sample quality for the CIFAR-10 dataset and consists of 2700 steps. For other datasets, the shape has less effect on the sample quality. See Figure A7B for the shape of the annealing schedule used.

We initialized the reverse chain on test images for the log-likelihood estimation and then sampled 10,000 intermediate distributions by using ten steps of HMC updates each. The temperature schedule is roughly exponential-shaped, and the reference distribution is an isotropic Gaussian. The variance in the estimation was generally less than 10% on the log scale. Due to the high variance in the results and to avoid getting dominated by a single outlier, we report the average of the log density instead of the log of the average density.

## Appendix E. Extended Samples and Inpainting Results

We provide more inpainting examples and further demonstrate the mixing during the sampling process in Figure A3. We also provide more samples for readers to visually judge the quality of our sample generation in Figure A4, Figure A5 and Figure A6. All samples are randomly selected.

## Appendix F. Sampling Process and Energy Value Comparison

Here we show how the average energy of samples behaves vs the sampling temperature. We also show an example of our model making out a distribution error that is common in most other likelihood-based models [54] Figure A7.
